# Alteration of the Cortical Actin Cytoskeleton Deregulates Ca^2+^ Signaling, Monospermic Fertilization, and Sperm Entry

**DOI:** 10.1371/journal.pone.0003588

**Published:** 2008-10-30

**Authors:** A. Puppo, Jong T. Chun, Giovanni Gragnaniello, Ezio Garante, Luigia Santella

**Affiliations:** Stazione Zoologica Anton Dohrn, Villa Comunale, Napoli, Italy; University of Oldenburg, Germany

## Abstract

**Background:**

When preparing for fertilization, oocytes undergo meiotic maturation during which structural changes occur in the endoplasmic reticulum (ER) that lead to a more efficient calcium response. During meiotic maturation and subsequent fertilization, the actin cytoskeleton also undergoes dramatic restructuring. We have recently observed that rearrangements of the actin cytoskeleton induced by actin-depolymerizing agents, or by actin-binding proteins, strongly modulate intracellular calcium (Ca^2+^) signals during the maturation process. However, the significance of the dynamic changes in F-actin within the fertilized egg has been largely unclear.

**Methodology/Principal Findings:**

We have measured changes in intracellular Ca^2+^ signals and F-actin structures during fertilization. We also report the unexpected observation that the conventional antagonist of the InsP_3_ receptor, heparin, hyperpolymerizes the cortical actin cytoskeleton in postmeiotic eggs. Using heparin and other pharmacological agents that either hypo- or hyperpolymerize the cortical actin, we demonstrate that nearly all aspects of the fertilization process are profoundly affected by the dynamic restructuring of the egg cortical actin cytoskeleton.

**Conclusions/Significance:**

Our findings identify important roles for subplasmalemmal actin fibers in the process of sperm-egg interaction and in the subsequent events related to fertilization: the generation of Ca^2+^ signals, sperm penetration, cortical granule exocytosis, and the block to polyspermy.

## Introduction

At the initial stage of fertilization, the interacting egg and sperm become mutually activated. Due to the availability of large quantities of gametes and the easiness of experimental manipulation, this early phase of the fertilization process has been extensively studied in echinoderms. In starfish, it has been clearly demonstrated that the fertilizing sperm undergoes an acrosome reaction in which the sperm head generates a very long tube-like structure called the ‘acrosomal process’ upon contacting the egg jelly coat [1]–[4]. The acrosomal process of the starfish sperm is particularly long, nearly 20 µm, which is well matched with the large size of the starfish egg (up to 200 µm in diameter) and provides a particularly useful opportunity for studying egg-sperm interactions [5], [6]. While the jelly coat of the egg stimulates the acrosome reaction of the sperm, the acrosomal process in turn activates the egg upon binding to its surface. The fusion of the gametes leads to a series of biochemical and morphological changes on both cells that are preceded by changes of membrane potential and massive release of intracellular Ca^2+^ [7]. The depolarization of the egg plasma membrane induced by the impact with the first successful sperm serves as a fast block against polyspermy [8]. On the other hand, the massive Ca^2+^ release stimulates exocytosis of cortical granules on the egg surface leading to the elevation of the egg vitelline layer. The formation of this so-called ‘fertilization envelope’ serves as a slow block against supernumerary sperm entry [9].

The massive and rapid release of Ca^2+^ in fertilized eggs has made it easier to study the general molecular mechanisms underlying Ca^2+^ mobilization inside the cell. Studies based on fluorescent Ca^2+^ indicators and with electrophysiological methods have demonstrated that the release of Ca^2+^ from intracellular stores is mediated by second messengers such as inositol 1,4,5-trisphosphate (InsP_3_), cyclic-ADP-ribose (cADPr), and nicotinic acid adenine dinucleotide phosphate (NAADP) [7], [10], [11]. Studies with caged second messengers photoliberated inside cells have demonstrated that each of the three second messengers induce characteristic Ca^2+^ waves [6], [12], [13]. In starfish eggs, the liberation of exogenous NAADP produces a sharp synchronized increase of Ca^2+^ signals in a narrowly defined subplasmalemmal domain [14]–[16]. At variance with NAADP, cADPR first induces Ca^2+^ liberation at several discrete spots inside the egg, which then merge to form a broad wave on the cortex which propagates to the center [17]. InsP_3_ may play a central role in the propagation of this Ca^2+^ release [18]. In mammalian eggs, blockade of the InsP_3_ receptor (InsP_3_R) with an antibody blocked the fertilization-induced Ca^2+^ oscillations, suggesting that InsP_3_ is instrumental in the generation of Ca^2+^ waves [19]. However, similar studies with anti-InsP_3_ antibodies in echinoderm eggs have not produced the same result [20]. In addition, an inhibitor of the InsP_3_ receptor, heparin, did not completely block the sperm-induced Ca^2+^ signals in echinoderm eggs [21]–[26]. In fertilized sea urchin eggs, the major increase in InsP_3_ production occurred after the initiation of the Ca^2+^ wave, raising the possibility that InsP_3_ might not be responsible for the initial surge of Ca^2+^ [27], [28]. Hence the Ca^2+^-releasing mechanism in echinoderm eggs appears to be fundamentally different from that of mammalian eggs with respect to the contribution of InsP_3_.

In starfish eggs, the different Ca^2+^-releasing second messengers have different roles. The rapid and cortex-restricted Ca^2+^ release by NAADP is reminiscent of the cortical Ca^2+^ flash induced by the fertilizing sperm [7]. On the other hand, the InsP_3_-evoked Ca^2+^ wave exhibits a more global mode of propagation. Comparison of Ca^2+^ signals induced by NAADP and by InsP_3_ in enucleated postmeiotic eggs has suggested that NAADP initiates the sperm-induced Ca^2+^ response, while the InsP_3_ is involved in the later propagation of the Ca^2+^ wave [7], [15], [29]. Intriguingly, the InsP_3_-evoked Ca^2+^ rise in enucleated eggs displayed comparable amplitude but a slightly delayed kinetics, and failed to trigger cortical granule exocytosis [15]. These observations raise the possibility that the InsP_3_-dependent Ca^2+^ release and the cortical granule exocytosis are under the regulation of unknown mechanisms influenced by nuclear factors such as MPF (Maturation Promoting Factor) [29].

We have recently observed that the actin cytoskeleton plays important roles in modulating intracellular Ca^2+^ release and cortical granule exocytosis in starfish eggs [30]–[33]. These observations have suggested that the actin cytoskeleton may play more than a structural role in these cells. Indeed, a number of observations have indicated that the role of the actin cytoskeleton is not limited to cell motility or cytokinesis, but extends to other different aspects of cell activity, e.g. to the regulation of ion channels and gene expression [34]–[36]. The actin cytoskeleton also plays pivotal roles in the fertilization process, as exemplified by its intervention in the acrosome reaction. In this communication, we show that fine regulation of the egg cortical actin network in starfish eggs is important for Ca^2+^ signaling and the formation of fertilization envelope, as well as for the control of monospermy and sperm entry.

## Materials and Methods

### Preparation of eggs and fertilization

Two species of starfish (*A. aranciacus* and *A. pectinifera*) were obtained from the Gulf of Naples, Italy and Mutzu Bay, Japan, respectively. Animals were maintained in circulating seawater at 16°C, and the gonads were dissected from the central dorsal area near the arms and transferred to cold, filter-sterilized seawater (FSW). Fully-grown immature oocytes were isolated as single cells by sieving through gauze several times in cold FSW. Free oocytes were isolated by repeated rinsing and low-speed centrifugation in cold FSW. To obtain postmeiotic eggs, immature oocytes were stimulated with 1-methyladenine (1-MA) as described previously [30]. For fertilization, 10 µl of ‘dry sperm’ collected from the male gonad were added to 1 ml of FSW, and then 3 µl of the diluted sperm were transferred to the incubation chamber containing postmeiotic eggs in 2 ml FSW.

### Microinjection, photoactivation of caged compounds and Ca^2+^ imaging

Microinjection of eggs was performed with an air-pressure Transjector (Eppendorf). In this standardized method, the amount of injected material was estimated at 1–2% of the egg volume. Hence, the final concentration of the injected material inside the eggs should have been 50 to 100-fold lower than the concentration in the injection pipette. The fluorescent calcium dye, Calcium Green 488, conjugated to 10 kDa dextran (Molecular Probes) was used in 5 mg/ml pipette concentration with the injection buffer (10 mM Hepes, pH 7.0, 100 mM potassium aspartate). The same injection buffer was used for delivering heparin (Sigma-Aldrich) and caged InsP_3_ (Molecular Probes) by microinjection. Caged InsP_3_ (5 µM pipette concentration) was co-injected with the fluorescent Ca^2+^ indicator into eggs. To activate the caged InsP_3_, oocytes were irradiated with 330 nm UV light for 25 sec with the use of a computer-controlled shutter system (Lambda 10-2, Sutter Instruments, Novato, CA, USA). Cytosolic Ca^2+^ changes were detected with a cooled CCD camera (MicroMax, Princeton Instruments, Inc., Trenton, NJ) mounted on a Zeiss Axiovert 200 microscope with a Plan-Neofluar 20×/0.50 objective. The quantified Ca^2+^ signal at a given time point was normalized to the baseline fluorescence (F_0_) following the formula F_rel_ = [F−F_0_]/F_0_, where F represents the average fluorescence level of the entire egg. Fluorescent Ca^2+^ images were analyzed with the MetaMorph Imaging System software (Universal Imaging Corporation, West Chester, PA, USA). The incubation conditions of jasplakinolide (JAS), latrunculin-A (LAT-A), and heparin are indicated in the figure legends. Unless specified otherwise, the control cells refer to the eggs from the same batches that were treated with the same vehicle for drug delivery.

### F-actin staining, laser-scanning confocal microscopy and transmission electron microscopy

F-actin was visualized by two methods, either with or without cell fixation. To visualize F-actin in living cells, starfish eggs were microinjected with 50 µM Alexa Fluor 488-conjugated phalloidin and visualized with confocal microscopy after 10 min incubation. To stain polymerized actin in fixed cells, starfish eggs were treated in 0.5% glutaraldehyde for 1 h and then permeabilized in 1% Triton X-100 (PBS) for 2 h before incubating in 0.1 µM of fluorescent phalloidin (1% Triton X-100, PBS) for 2 h. After extensive washing in PBS, eggs were transferred to an experiment chamber and were observed with an Olympus Fluoview 200 laser-scanning microscope with a 60× (1.20 NA) objective. Images of F-actin stained with Alexa Fluor 488-conjugated phalloidin were recorded through a BP 510–540 emission filter. Transmitted light and fluorescent confocal images of the control and the experimental eggs were acquired from the equivalent cytoplasmic planes. For transmission electron microscopy, oocytes were first fixed in 1% glutaraldehyde (FSW, pH 8.0) for 1 h at room temperature and then rinsed extensively in FSW before being treated 1 h with FSW containing 1% osmium tetroxide. Specimens were dehydrated in increasing concentrations of alcohol and embedded in EPON 812. Sections were stained with 2% uranyl acetate and 0.2% lead citrate and examined with a LEO 912 AB energy filter transmission electron microscope.

## Results

### The sperm induces Ca^2+^ waves and the fertilization envelope before its head is incorporated into starfish eggs

**Figure 1:**
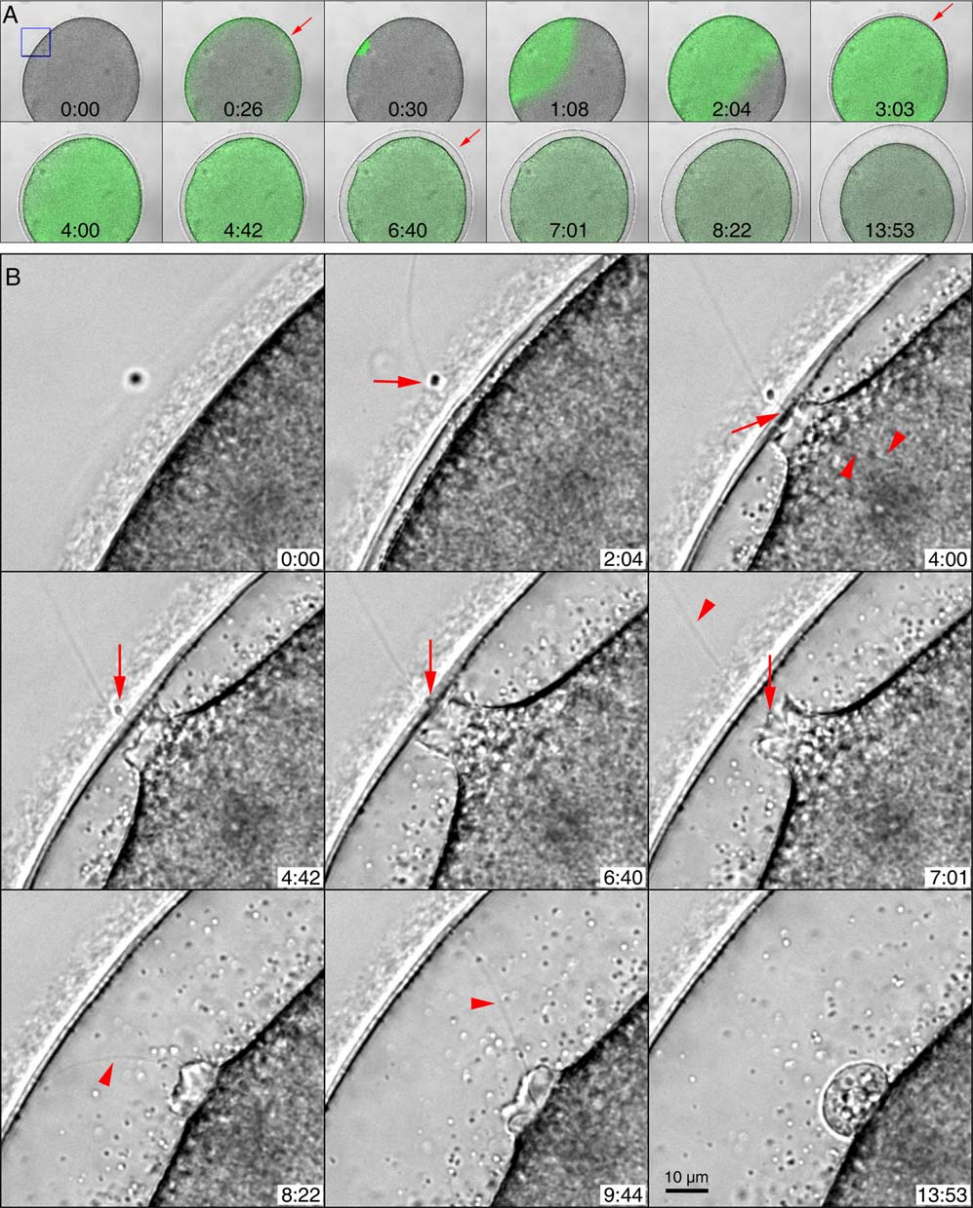
The spatiotemporal relationship among sperm entry, Ca^2+^ release, and the elevation of the vitelline layer in fertilized starfish eggs. (A) The transmission images of a representative fertilized egg (*A. aranciacus*) were superimposed with the corresponding fluorograms of the Ca^2+^ indicator at each time point. The moment of sperm's arrival at the jelly coat was set to t = 0:00 (min:sec). Following the quick cortical flash at 0:26 (arrow), a massive Ca^2+^ wave initiated from the sperm entry site (0:30) and propagated to the opposite side of the egg. At 3:03 when the Ca^2+^ wave had already encroached upon the entire cytoplasm, the fertilization envelope began to be elevated (arrow). The fertilization envelope was fully formed only after the Ca^2+^ wave had traversed the entire cytoplasm at 6:40 (arrow). (B) Detailed views of the sperm entry site (the area marked by a small blue rectangle in panel A) during fertilization. At 2:04 when the vitelline layer is locally elevated, the sperm is still located in the jelly coat (arrow). At 4:00, the sperm head still remains on the outside surface of the egg, but the long acrosomal process (arrow) is inside the egg and connected to a filamentous structure (arrowheads). At 4:42, the sperm head is still visible (arrow), the vitelline layer is further elevated and the focal plasma membrane at the sperm entry site is now being detached from the vitelline layer. At 4:42 and at 6:40, the sperm head (arrow) is still inside the jelly coat. At 7:01, the sperm head is inside the egg cytoplasm (arrow), and the plasma membrane is retracting behind the sperm. The tail is still outside (arrow). At 8:22 and at 9:44, the tail of the sperm finally enters the egg cytoplasm (arrowhead), as the plasma membrane further seals to form a fertilization cone. The motion picture of the entire process is available as a video file (Data S1).

The fertilizing spermatozoon induces a massive release of Ca^2+^ inside the egg, which leads to the exocytosis of cortical granules and to the subsequent elevation of the vitelline layer [7], [12], [37], [38]. The precise spatiotemporal relationship that exists for sperm entry, Ca^2+^ entry, and the formation of the fertilization envelope has not been described in detail. Owing to the large cell size and optical transparency of the cytoplasm, starfish eggs have been an excellent model system for studying intracellular Ca^2+^ release and morphological changes with the help of fluorescent Ca^2+^ indicators and imaging systems [6]. As shown in Fig. 1A, sperm addition to the *A. aranciacus* egg produced the characteristic cortical Ca^2+^ flash and the propagation of the Ca^2+^ wave from the site of sperm-egg interaction. We have noticed that the fertilizing sperm is able to induce Ca^2+^ signaling and to initiate the vitelline layer elevation while still outside the egg (arrow in Fig. 1A, at 3:03). One minute after the initiation of the Ca^2+^ burst, the vitelline layer was further elevated, but the sperm was still outside the egg. However, the sperm had inserted its long acrosomal process in the egg cytoplasm (arrow in Fig. 1B, at 4:00), forming a fertilization cone. Curiously, the acrosomal process appeared prolonged inside the egg by the conjunction with filamentous structure inside the egg (arrowheads in Fig. 1B, at 4:00). The vitelline layer became further elevated with time, while the sperm was still located in the jelly coat outside the egg. At this point, the plasma membrane of the egg was not yet detached from the vitelline layer in the fertilization cone. Only after this portion of the egg plasma membrane had become detached from the elevating vitelline layer did the sperm physically enter the egg cytoplasm (Fig. 1B, at 7:01). At 4:42 the sperm head was still visible as it approached the egg plasma membrane (arrow). After it had crossed it (at 7:01) it became lost among the other cytoplasmic densities (arrow), only its tail still being visible outside (arrowhead). As the vitelline layer was fully elevated, the sperm tail also entered the egg cytoplasm (Fig. 1B, at 8:22 and at 9:44, arrowheads). At the conclusion of the process, the egg plasma membrane at the sperm entry site sealed with the formation of a round bleb (Fig. 1B, at 13:53). The entire process is presented as a video file in Data S1.

### Heparin induces polyspermy and delays the propagation of the sperm-induced Ca^2+^ wave inside the fertilized egg

**Figure 2:**
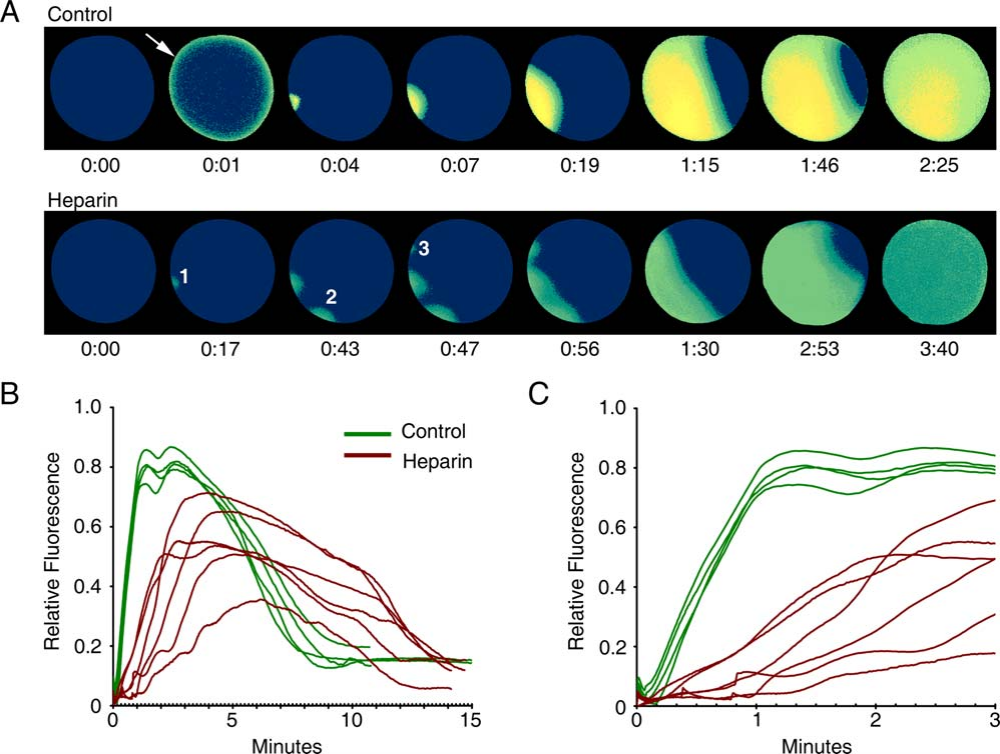
Heparin induces polyspermy and impedes the propagation of the sperm-induced Ca^2+^ wave in fertilized starfish eggs. (A) Relative fluorescence pseudo-colored images of the Ca^2+^ indicator depicting the propagating patterns of Ca^2+^ waves in the presence or absence of heparin (25 mg/ml, pipette concentration). The moment of the first detectable Ca^2+^ signal was set to t = 0 in both cases. In heparin-treated eggs, the cortical flash seen in control eggs (arrow) was absent, and polyspermy was evident in all cases (n = 6). The initial Ca^2+^ spots representing polyspermy were numbered in order of occurrence. (B) Quantification of intracellular Ca^2+^ levels in fertilized eggs in the control (green curves, n = 4) and heparin-injected (brown curves, n = 6) eggs. (C) The initial phase of the same Ca^2+^ rise depicted in (B) was plotted in smaller time scale in order to demonstrate the slowed kinetics of the Ca^2+^ rise in heparin-treated eggs.

**Figure 3:**
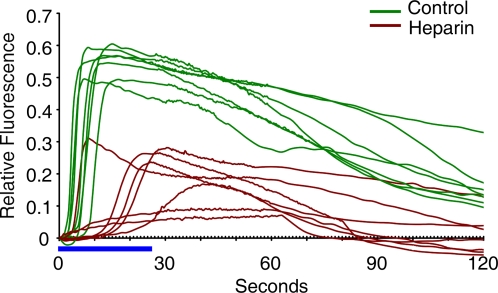
Inhibition of InsP_3_-dependent Ca^2+^ release by heparin. Photoactivation of the caged InsP_3_ (10 µM, pipette concentration) inside *A. aranciacus* eggs produced massive release of Ca^2+^ from intracellular stores (green curves, n = 7). In eggs pre-injected with heparin (25 mg/ml, pipette concentration), the Ca^2+^ response was significantly reduced in its amplitude, but not completely abolished (brown curves, n = 7). The duration of the UV illumination was marked by the blue bar.

Heparin has been used extensively as an antagonist of InsP_3_Rs. As has been reported for *Xenopus* and sea urchin eggs [24], [25], the microinjection of heparin into starfish eggs also increased the occurrence of polyspermy. In heparin-treated eggs, we found that Ca^2+^ waves evidently initiated at multiple sperm-egg interaction sites (Fig. 2A, lower panel). The average number of detectable initial Ca^2+^ spots in heparin-treated eggs was 4.8 after fertilization, as opposed to 1.2 in the control eggs (n = 5, each case). In addition, the characteristic cortical flash seen in the control egg was abolished in the heparin-treated eggs. The amplitude and kinetics of the intracellular Ca^2+^ rise were also significantly reduced (Fig. 2B). The Ca^2+^ transients peaked at 0.55±0.12 arbitrary units within 272 sec in heparin-treated eggs, as opposed to 0.83±0.03 arbitrary units in 70 sec in the monospermic control eggs. The delayed kinetics and lowered amplitude of the Ca^2+^ peak in heparin-treated eggs are not due to polyspermy itself, because occasional polyspermy occurring in control eggs displayed an even faster Ca^2+^ rise (not shown). Considering that heparin is an inhibitor of InsP_3_ receptors, the inhibition of Ca^2+^ propagation was not surprising. The treatment with heparin indeed inhibited the Ca^2+^-releasing activity of injected InsP_3_. The average amplitude of the Ca^2+^ peak seen in control eggs (0.55±0.04 arbitrary units) was strongly reduced (0.25±0.05), but not completely abolished (Fig. 3). The significance of this observation will be discussed in more detail later on (see Discussion).

### Heparin may influence intracellular Ca^2+^ release in different mechanisms

**Figure 4:**
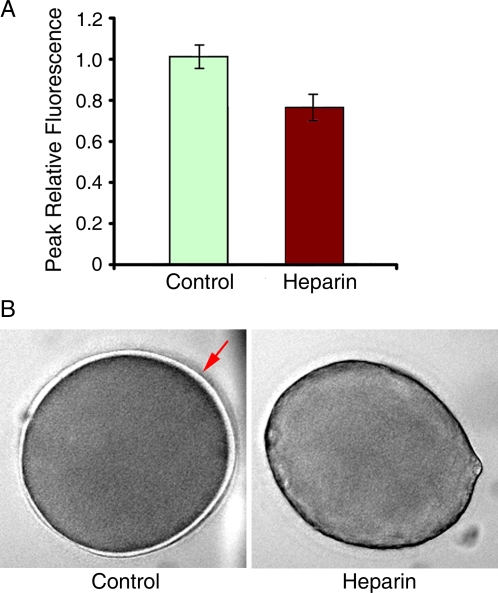
Heparin partially inhibits cADPr-dependent Ca^2+^ release. (B) Activation of the caged cADPr pre-injected in *A. aranciacus* eggs results in intracellular release of Ca^2+^. The altitude of the Ca^2+^ peak was significantly reduced in heparin-treated eggs compared to the control (n = 7, in each case). (B) Transmission photomicrographs of control and heparin-treated eggs following cADPr uncaging (450 µM, pipette concentration) and subsequent Ca^2+^ release. The typical elevation of the vitelline layer seen in the control eggs (arrow) was absent in the eggs treated with heparin.

As a specific inhibitor of InsP_3_ receptors, heparin should not interfere with Ca^2+^ release through the cADPr-gated ryanodine receptors. However, we found that Ca^2+^ release in starfish eggs by cADPr photoliberated from injected caged cADPr was substantially reduced by heparin. The peak values of 1.01±0.11 arbitrary units in the controls were reduced to 0.76±0.12 (Fig. 4A). Although cADPr uncaging inside the heparin-treated eggs still evoked a substantial Ca^2+^ release, the mobilization of Ca^2+^ failed to elevate the vitelline layer as seen in the control eggs (Fig. 4B, arrow). Taken together, these observations suggested that heparin may also influence cell elements other than InsP_3_ receptors, thereby interfering with the intracellular Ca^2+^ signaling in a more general way.

**Figure 5:**
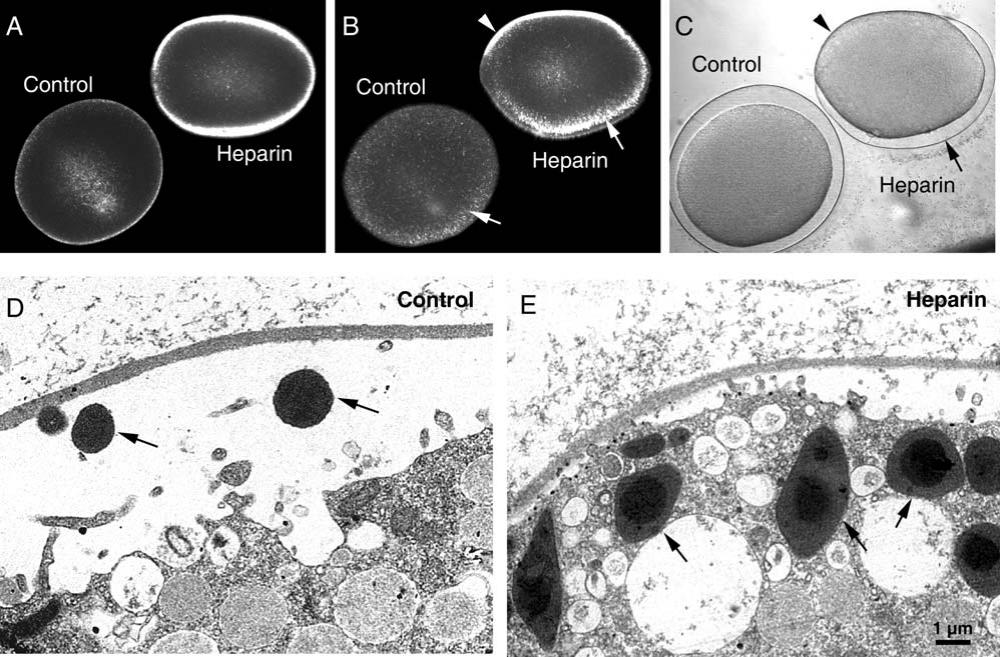
Heparin alters the cortical actin cytoskeleton and interferes with the formation of the fertilization envelope. (A) The actin cytoskeleton was visualized in living cells by use of fluorescent phalloidin. Mature eggs pre-injected with buffer (control) or heparin (25 mg/ml) were microinjected with fluorescent phalloidin to visualize F-actin. It is evident that heparin induced hyperpolymerization of the cortical actin. (B) The same eggs were fertilized with sperm. In the control egg, the tight cortical actin network dispersed 9 min after the addition of sperm (arrow). The same trend was also evident in one part of the heparin-treated egg (arrow). (C) The control egg displayed normal formation of the fertilization envelope. In the heparin-treated egg, the formation of the fertilization envelope was blocked on the side where the tight cortical actin network did not disperse (arrowhead). (D) In the subplasmalemmal regions of the fertilized eggs visualized by electron microscopy, the images of the cortical granule cores (arrows) are occasionally captured in the perivitelline space of the control eggs. In contrast, heparin-treated eggs exhibited a pile of cortical granules (arrows) in the subplasmalemmal region (E).

We have recently demonstrated that heparin hyperpolymerizes cortical actin in premeiotic oocytes of starfish [33]. We have now found that heparin dramatically increased actin polymerization in the subplasmalemmal region of the postmeiotic eggs as well. Mature eggs of *A. aranciacus* microinjected with heparin displayed much denser phalloidin labeling in the cortical actin layer (Fig. 5A). This remarkable enhancement of actin polymerization in the cortex is not due to a potential secondary effect of phalloidin itself, which has been known to stabilize F-actin [39]. Glutaraldehyde fixation of the heparin-treated eggs before visualizing actin with phalloidin also exhibited heparin-specific hyperpolymerization of cortical actin (Data S2). The intricate network of actin filaments in the control cortex dispersed soon after fertilization (Fig. 5B). This trend of F-actin to become reorganized was strong enough to be evident even in the major part of the heparin-treated eggs after fertilization (Fig. 5B, arrow). However, the heparin-induced hyperpolymerization of actin prevented the centripetal movement of the actin fibers to the other part of the egg (Fig. 5B, arrowhead). The sperm interaction often failed to elevate the vitelline layer on the entire surface of the heparin-treated eggs, resulting in a partial and polarized formation of the fertilization envelope (Fig. 5C; 6 out of 10 eggs). The suggestion that reorganization of the cortical actin cytoskeleton facilitated the exocytosis of cortical granules was reinforced by the finding that the fertilization of heparin-treated eggs produced elevation of the vitelline layer only in the area from which the cortical actin network had dispersed (Fig. 5B and C, arrows). The regional blockade of the cortical granule exocytosis by heparin was also visualized by electron microscopy. Whereas the control egg exported the dark shaded cortical granules from the cytoplasm (Fig. 5D), the heparin-treated egg conspicuously accumulated them in the subplasmalemmal region adjacent to the partially elevated fertilization envelope (Fig. 5E).

### Alteration of the cortical actin cytoskeleton induces abnormal and polyspermic formation of fertilization cones

**Figure 6:**
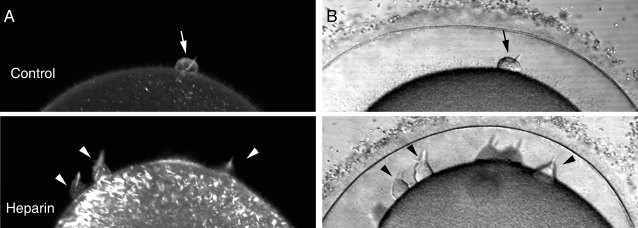
Heparin induces polyspermy and abnormal formation of fertilization cones. Formation of the fertilization cone was monitored in living eggs after sperm addition. (A) F-actin was visualized by microinjection of fluorescent phalloidin. (B) Transmission photomicrographs of the same eggs. Both phalloidin-stained actin networks and the transmission photomicrographs displayed formation of single and round fertilization cone (arrow) in the control egg by 9 min after adding sperm. In contrast, heparin-treated eggs displayed abnormal formation of multiple and piriform-shaped fertilization cones (arrowheads).

**Figure 7:**
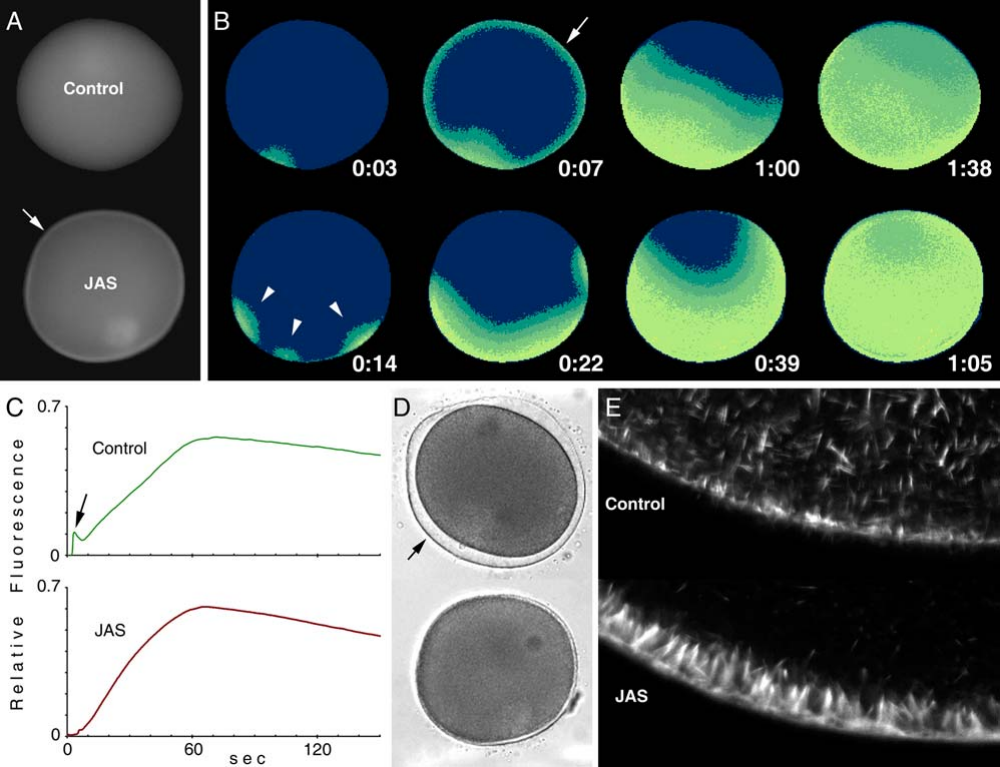
Effects of the actin-polymerizing agent jasplakinolide (JAS) on fertilization. (A) Mature eggs of *A. aranciacus* were injected with Ca^2+^ dye and incubated in the presence or absence of JAS (12 µM for 20 min). The moment of the first detectable Ca^2+^ release was set to t = 0:00 (min:sec). Conspicuous accumulation of Ca^2+^ dyes was evident in the submembraneous zones of the JAS-incubated eggs (arrow). (B) At 0:07, the control eggs manifested the cortical flash of Ca^2+^ (arrow), which is absent in the JAS-incubated eggs. Instead, JAS induced polyspermy and produced multiple initiation sites of Ca^2+^ signals at 0:14 (arrowheads). (C) Quantification of intracellular Ca^2+^ levels in the control and the JAS-incubated eggs after the addition of sperm. The arrow represents the cortical flash that is absent in the JAS-incubated eggs. (D) The formation of the fertilization envelope seen in the control eggs (arrow) is totally blocked in the JAS-incubated eggs. (E) Comparison of the cortical actin networks in the control and JAS-incubated eggs (before fertilization) using fluorescent phalloidin. In the presence of JAS, starfish eggs displayed remarkable actin hyperpolymerization in the subplasmalemmal region. In contrast, actin fibers in the inner cytoplasm were often reduced by JAS, reflecting the depletion of monomeric actin pool inside the cell [40].

Since heparin induced polyspermy, we examined how it affected the formation of fertilization cones in starfish eggs. In the heparin-treated eggs loaded with phalloidin, the average number of fertilization cones was 6.4 per cell (n = 10), while it was 1.6 (n = 10) in the control eggs. Although the fertilization cones in both cases were rich in F-actin (Fig. 6A, arrow and arrowheads), the heparin-treated eggs exhibited a substantial concentration of actin fibers in the cortical domain underneath the fertilization cones (Fig. 6A). Furthermore, the conical shape of the fertilization cones in heparin-treated eggs (Fig. 6, arrowheads) was at variance with that of control eggs, which resembled a round bleb (Fig. 6, arrows). These fundamental differences in number and shape of fertilization cones were not due to the presence of phalloidin, as similar transmission light microscopy experiments without phalloidin produced the same results (not shown). A plausible concern at this point regards the way heparin induced polyspermy and abnormal formation of the fertilization cones, i.e., whether it did so by inhibiting the InsP_3_R, or by alterating the actin cytoskeleton at the subplasmalemmal region of the egg [33]. To test whether polyspermy and the changes of Ca^2+^ signaling were linked to the alteration of the cortical actin cytoskeleton, we examined the effect of jasplakinolide (JAS), a well characterized agent that stimulates cortical actin polymerization [40]. As was the case for heparin, fertilization of the starfish eggs pre-incubated with JAS produced polyspermy, as indirectly shown by the presence of multiple initiation sites of Ca^2+^ waves that represent loci of sperm interaction (Fig. 7B, arrowheads). The most evident effect of JAS was the elimination of the cortical flash of control eggs (Fig. 7B, arrow at 0:07). This effect is evident in the quantified Ca^2+^ curves, where the small peak at the very beginning of the fertilization-induced Ca^2+^ rise was blocked by JAS (Fig. 7C, arrow). On the other hand, the overall pattern of the Ca^2+^ waves at later stages of fertilization was virtually the same as in the control egg. However, despite the massive release of Ca^2+^ that reached the cortex of the cell, JAS completely blocked the vitelline layer elevation seen in the control egg (Fig. 7D, arrow). An additional difference between the JAS-incubated and the heparin-treated eggs is that the former produced no fertilization cone despite the initial polyspermic interactions and the multiple foci of local Ca^2+^ release (Fig. 7B), as the formation of the fertilization cone is associated with vitelline layer elevation (Fig. 1B). F-actin visualized by phalloidin became dramatically concentrated, corroborating the suggestion that all these changes on the egg surface are linked to the alteration of the cortical actin cytoskeleton in the subplasmalemmal region of the JAS-treated eggs (Fig. 7E).

### Alteration of the cortical actin cytoskeleton blocks sperm entry

**Figure 8:**
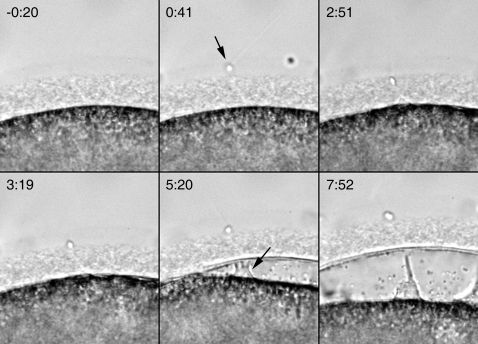
Heparin blocks sperm entry. The fertilization process in the heparin-injected egg was monitored with a CCD camera, and the key moments were presented by still-shot photomicrographs. The moment of sperm attachment to the egg surface was set to t = 0:00 (min:sec). At 0:41, the sperm was still attached to the jelly coat (arrow). Afterwards, the sperm attempts but fails to penetrate the jelly coat. At 5:20, the vitelline layer is visibly elevated, but the sperm is still completely outside the jelly coat. The formation of the fertilization cone is evident under the elevating membrane (arrow). At 7:52, the vitelline layer is further elevated, but the fertilization cone fails to pull in the sperm head. The fertilization envelope is now being established while the sperm is still outside. The motion picture of the entire process is available as a video file in Data S3.

As mentioned earlier, alteration of the cortical actin cytoskeleton by heparin leads to the polyspermic formation of fertilization cones. To test if heparin also affected sperm entry, the fertilization process was monitored with a CCD camera. Although heparin-treated eggs produced at least 5 times more fertilization cones than control eggs in a given focal plane, the frequency of actual sperm entry through these fertilization cones was significantly lower. Whereas 9 out of 10 fertilization cones in the focal plane of control eggs exhibited successful sperm entry, the success rate in the heparin-treated eggs was 14 out of 28 (n = 8). Similar results were obtained when sperm were prestained with the DNA dye Hoechst 33342 (not shown). The increased occurrence of ‘empty’ fertilization cones in heparin-treated eggs enabled us to monitor the abortive procedure of sperm entry. In the heparin-treated eggs, sperm initially penetrated the jelly coat (Fig. 8, see the position of the sperm head at 0:41 and at 2:51) in a way similar to the control, but the continuous movement of the elevating vitelline layer failed to ‘pull’ the sperm into the egg cytoplasm. The vitelline layer continued to elevate with the formation of the ‘empty’ fertilization cones (Fig. 8, arrow at 5:20), but in most cases the sperm was apparently pushed back to the jelly coat (see the video file in Data S3).

### Alteration of the actin cytoskeleton affects the Ca^2+^ release pattern and cortical granule exocytosis in starfish eggs

**Figure 9:**
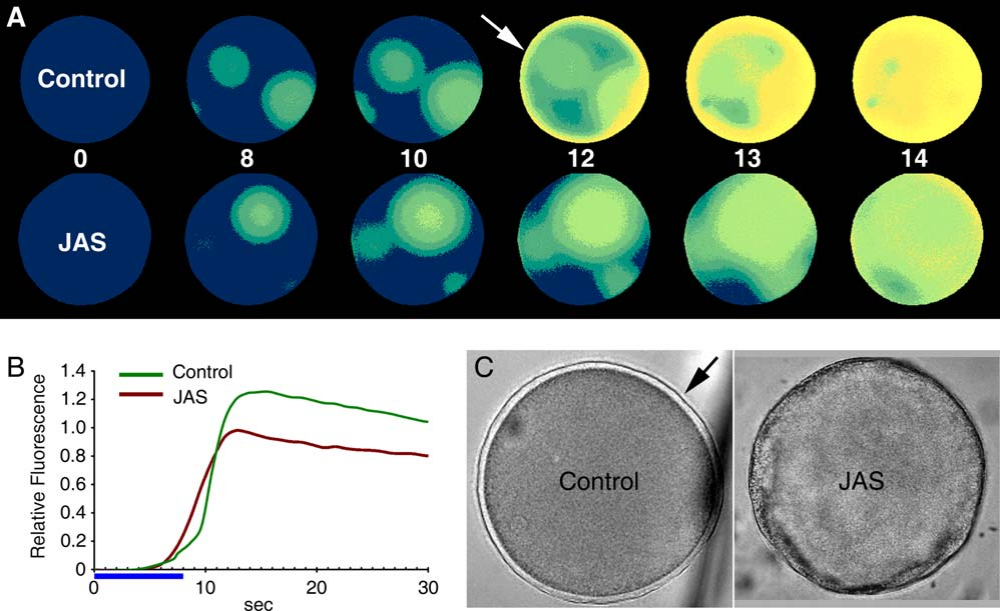
Effects of the actin-depolymerizing agent JAS on cADPr-dependent Ca^2+^ mobilization and the elevation of the vitelline layer. Mature eggs of *A. aranciacus* loaded with Ca^2+^ dye and caged cADPr were illuminated with UV to activate the caged second messenger. (A) Relative fluorescence images of the Ca^2+^ indicator depicting the propagating patterns of Ca^2+^ waves in the presence or absence of JAS (6 µM). The photoactivation of cADPr initiated Ca^2+^ release from multiple sites and produced the characteristic cortical Ca^2+^ signals (arrow) in the control eggs. The strong cortical Ca^2+^ release seen in the control was absent in the JAS-incubated eggs. (B) Comparison of the intracellular Ca^2+^ release in the control (green curve) and JAS-incubated eggs after fertilization (brown curve). (C) Photoactivation of cADPr and subsequent release of Ca^2+^ elevated the vitelline layer in control eggs (arrow). The elevation of the vitelline layer was blocked in the JAS-incubated eggs.

**Figure 10:**
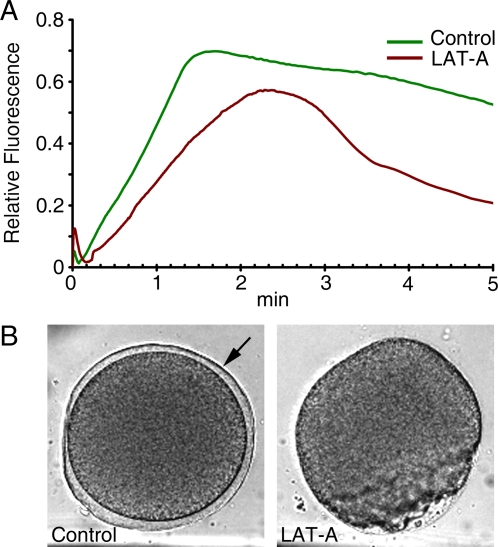
Effects of the actin depolymerizing agent latruncunlin A (LAT-A) on fertilization. Mature eggs of *A. pectinifera* were injected with Ca^2+^ dye and incubated with or without LAT-A (3 µM 30 min) before fertilization. (A) Intracellular Ca^2+^ release during fertilization. LAT-A slightly lowered the Ca^2+^ release during fertilization, but the cortical flash (the small initial Ca^2+^ peak) in the LAT-A-treated eggs (brown curve) was evidently enhanced in comparison with that of the control eggs (green curve). (B) The elevation of the vitelline layer seen in the control eggs (arrow) was completely blocked during the fertilization of the LAT-A treated eggs.

Since heparin not only inhibited InsP_3_ receptors but also induced actin hyperpolymerization (Fig. 5), the cause for the repression of Ca^2+^ signaling in fertilized eggs pretreated with heparin (Fig. 2) is not straightforward. The finding that heparin, which is not known to interfere with ryanodine receptors, substantially inhibited the Ca^2+^ release by cADPr (Fig. 4), supported the idea that changes of the actin cytoskeleton may contribute to the Ca^2+^ release process. To further test this idea, we have used other agents that either polymerize or depolymerize actin filaments. The consequence of the actin hyperpolymerization by JAS has been already mentioned in the context of fertilization (Fig. 7). As shown in Fig. 9A, the cADPr-induced Ca^2+^ release was also significantly affected by JAS. Liberation of uncaged cADPr in control and JAS-treated eggs gave rise to Ca^2+^ release from several spots. However, the massive and global release of Ca^2+^ characteristically seen in the cortex of control eggs (Fig. 9A, arrow) was much compromised in the JAS-treated ones. As a whole, the amplitude of the cADPr-evoked Ca^2+^ signal was substantially lowered by JAS (Fig. 9B). However, the Ca^2+^ wave eventually propagated to the cortex; and with this comparably massive Ca^2+^ signaling, cortical granule exocytosis should have occurred. Nonetheless, the vitelline layer elevation, which results from the cortical granule exocytosis in the control eggs (Fig. 9C, arrow), was totally missing in JAS-treated eggs. These results suggest that the enhancement of F-actin content in the cortex is linked to the inhibition of cortical Ca^2+^ release and to the blockade of cortical granule exocytosis. Similar experiments with the actin-depolymerizing agent LAT-A led to slightly different effects upon Ca^2+^ signaling, but had the same effect on vitelline layer elevation seen on the JAS-incubated eggs (Fig. 10A). LAT-A led to diminished Ca^2+^ signals during the fertilization of *A. pectinifera* eggs. However, the characteristic cortical flash was significantly increased in LAT-A-treated eggs, suggesting that the decreased cortical layer of polymerized actin [33] was more permissive toward the generation of the cortical flash. On the other hand, the elevation of vitelline layer was totally blocked in LAT-A-treated eggs as well, indicating that not only the hyperpolymerization but also the hypopolymerization of cortical actin had a negative impact on the process of cortical granule exocytosis (Fig. 10B).

## Discussion

Signaling through Ca^2+^ and its binding proteins plays important roles in nearly all cell activities [18], [41], [42]. Mobilization of Ca^2+^ to modulate the targets of its signaling function inside living cells is most easily observed in eggs during their interaction with a fertilizing spermatozoon. The intracellular release of Ca^2+^ can occur in the form of a single wave or of repetitive Ca^2+^ spikes depending on the egg species [6], [43]. The generation and the propagation of the Ca^2+^ wave have been studied in detail, as a number of Ca^2+^-evoking second messengers have been identified [44]. The best known of these second messengers is InsP_3_ which releases Ca^2+^ by acting on a specific receptor/channel in the endoplasmic reticulum. The conventional inhibitor of the InsP_3_ receptor is heparin, which has been widely used to assess the InsP_3_-Ca^2+^ pathway. To our surprise, we have observed that heparin also induces actin hyperpolymerization in the subplasmalemmal region of the starfish egg [33]. This unexpected result was specific for heparin, since a structural analog was ineffective (Data S2). As the effect of heparin on the cortical actin cytoskeleton is inseparable from the inhibition of InsP_3_ receptors, we decided to revaluate the experimental data obtained from heparin-treated eggs.

As expected from earlier work in *Xenopus* and sea urchin eggs [24], [25], preinjection of *A. aranciacus* eggs with heparin severely affected the generation of the sperm-induced Ca^2+^ signals but did not completely block them. Intriguingly, heparin also did not completely block the Ca^2+^ signals evoked by exogenously introduced InsP_3_ in starfish eggs. This result was surprising as the amount of heparin we used in our experiment [25 mg/ml (pipette concentration)] was nearly 5 times higher than the dose that would completely block the Ca^2+^ channel activity of InsP_3_ receptors in isolated microsomes [45]. As for the amount of InsP_3_ introduced into the cell, this was two fold lower than the levels of endogenous InsP_3_ measured under physiological conditions at the height of the Ca^2+^ liberation peak [27]. The partial repression of the Ca^2+^ signal could have been attributed to the possibly insufficient efficacy of heparin as an InsP_3_R inhibitor inside a cell as large as the starfish egg. However, it appears more logical to assume that the InsP_3_ receptors were completely repressed by heparin as they would have been *in vitro* [45]. If this is the case, InsP_3_ might also liberate Ca^2+^ inside the egg by a mechanism independent of the InsP_3_Rs. Alternatively, and perhaps more plausibly, heparin may affect the generation of Ca^2+^ signals by a mechanism additional to the inhibition of InsP_3_R. The finding described here showing that heparin substantially inhibited the cADPr-evoked Ca^2+^ signals (which was in line with earlier reports from sea urchin eggs [46], [47]) argues in favor of this alternative possibility. The working hypothesis formulated at this point was that the portion of Ca^2+^ liberation not linked to the action of InsP_3_Rs was somehow related to the cytoskeletal actin changes induced by heparin.

In sea urchin and starfish eggs, heparin affects the electrical property of the plasma membrane. Hence, heparin-induced changes of the activation current at fertilization [25], [48] may have caused the increased frequency of polyspermy, which is an established effect of heparin, as a result of the failed ‘electrical block.’ The fast blockade of supernumerary sperm entry by this mechanism of fertilization membrane depolarization has been also reported in starfish eggs [5]. While this mode of prevention of polyspermy has been controversial [8], [49], heparin may also induce polyspermy by a long-term mechanism based on structural changes of the egg surface. Indeed, our studies have shown that in normal fertilization, eggs displayed a round fertilization cone at the site of sperm penetration which initiated a series of structural changes that blocked the interaction of additional sperms on the egg surface. In the heparin-treated eggs, the contact with the sperm induced the formation of multiple and abnormally shaped fertilization cones, showing that a process leading to monospermy had become deregulated at the initial stage of egg-sperm interactions.

The idea that the heparin-induced polyspermy was linked to the structural alteration of the cortical actin cytoskeleton was strongly supported by the finding that cortical actin hyperpolymerization by JAS also led to polyspermy, as judged by the formation of multiple initiation sites of Ca^2+^ signals. Although heparin and JAS have similar effects on the hyperpolymerization of cortical actin, the two agents have different effects on the formation of the fertilization cone and on the elevation of the fertilization envelope. The role of the actin cytoskeleton in the proper progression of cortical granule exocytosis has been demonstrated in the eggs of several animal species [33], [50].

Perhaps the difference between heparin and JAS in these processes may reflect qualitative differences in the way actin is hyperpolymerized. The analyses of time-lapse images (Fig. 1) and the motion picture of the fertilization process at the sperm interaction sites (Data S1) suggest that the swelling of the perivitelline space might be a mechanism to incorporate the sperm into the egg. However, the elevating fertilization envelope in heparin-treated eggs often failed to bring the sperm into the fertilization cone. Thus, in these abortive fertilization cones, sperm may have not been correctly anchored to the structural element that mediates its penetration. The filamentous structure connected to the acrosomal process inside the control eggs (Fig. 1B, at 4:00, arrowheads) might be the anchorage point for sperm entry. At fertilization, the cones are filled with microfilaments that are continuous with the actin filaments in the egg cortex [51], which have been shown to play essential roles in sperm entry in starfish and several other animal species [4], [52]–[57]. How they actually regulate monospermy is a different and still unsolved problem. Our data suggest that the actin cytoskeleton may contribute to monospermy at two checkpoints: (1) by restricting the number of sperm-egg interaction sites on the egg surface, (2) by transmitting the dynamic rearrangement of cortical actin fibers to the entire egg sphere. The facilitated exocytosis of cortical granules by the restructuring and translocating actin fibers at the entire egg surface is eventually likely to serve as a slow block to polyspermy.

The results in this study corroborate our previous findings that the actin cytoskeleton in starfish eggs plays a regulatory role in shaping Ca^2+^ signals [16], [30]–[33]. These observations had suggested that intracellular Ca^2+^ signaling in the cortex was significantly influenced by the polymerization status of the local actin filaments, i.e., actin hypopolymerization enhanced Ca^2+^ signaling. Accordingly, starfish eggs preinjected with the actin-depolymerizing protein, cofilin, displayed significantly increased Ca^2+^ signals in response to the Ca^2+^-mobilizing second messengers InsP_3_ and NAADP, and to sperm [31]. Whereas it has been established that Ca^2+^ signals remodel the actin cytoskeleton [58], [59], the mechanism(s) by which the actin cytoskeleton affects Ca^2+^ signaling is still unclear. InsP_3_ receptors, however, have been shown to be linked to actin filaments that regulate their function and subcellular distribution [60], [61]. Thus, the dynamic restructuring of the actin cytoskeleton might facilitate or inhibit the Ca^2+^ release process from the InsP_3_ dependent internal stores. Such a regulatory role for actin filaments has been directly demonstrated for both Ca^2+^ influx and the ligand-gated Ca^2+^, release from the ER [62]–[65]. Alternatively, the actin cytoskeleton itself could serve as a reservoir of Ca^2+^ which would be released when actin filaments are severed or depolymerized [30], [33], [66], [67]. That F-actin binds Ca^2+^ was also indirectly shown by the increased fluorescence of Ca^2+^ dyes in the cortex of eggs treated with JAS (Fig. 7A).

In summary, we have demonstrated that the alteration of the cortical actin cytoskeleton in the starfish eggs leads to the deregulation of monospermic sperm interaction, of Ca^2+^ signaling, of cortical granule exocytosis, and of the sperm entry process. Our studies using heparin have shown that the intracellular translocation of F-actin was directly correlated with the local elevation of the vitelline layer in response to sperm, suggesting that the actin cytoskeleton may play a crucial role in modulating cortical granule exocytosis. The demonstration of the physiological roles played by the actin cytoskeleton in nearly all aspects of fertilization process suggests that a strategy employing actin filaments is common to both sperm and eggs.

Although the main focus of our work was on the role of the cortical egg cytoskeleton in the fertilization process, it has also produced a finding that is very significant for our general understanding of the fertilization mechanism. This was the visualization of the single sperm fertilizing the egg. The sperm set in motion a series of essential events in the egg cytoplasm such as the generation of the Ca^2+^ signals and restructuring of the cortex. All these events take place while the sperm is still outside the egg, contacting the egg plasma membrane only with its long acrosomal process. This demonstration bears significant implications as to the spatial and physicochemical interplays between the sperm and the egg during the initial stages of the fertilization process.

## Supporting Information

Data S1 The video animation of the fertilization process in *A. aranciacus*, which was described in detail in the format of still-shot photos in Fig. 1B of the manuscript. Sperm incorporation is seemingly driven by the ‘elastic force’ of the cortical cone that is being detached from the plasma membrane.
(6.12 MB AVI)

Data S2 Heparin hyperpolymerizes cortical actin. A. aranciacus oocytes were treated with 1-MA for 1 h to induce meiotic maturation, and then microinjected with either buffer (A), heparin (B), or De-N-sulfated heparin (C). Following cell fixation with glutaraldehyde, F-actin was stained with fluorescent phalloidin. The hyperpolymerization of cortical actin seen in heparin-treated eggs (B) was not evident in the eggs pre-injected with the structural analog of heparin (C).
(0.99 MB TIF)

Data S3 The video animation of the failed sperm entry in heparin-treated eggs of *A. aranciacus*. The still-shot images of this movie clip were described in detail in Fig. 8.
(2.81 MB AVI)
