# Supplementary figures and images for: Alteration of the Cortical Actin Cytoskeleton Deregulates Ca2+ Signaling, Monospermic Fertilization, and Sperm Entry
Source: PLoS One. 2008 Oct 30;3(10):e3588. doi: 10.1371/journal.pone.0003588 (PMC2570615; doi:10.1371/journal.pone.0003588)

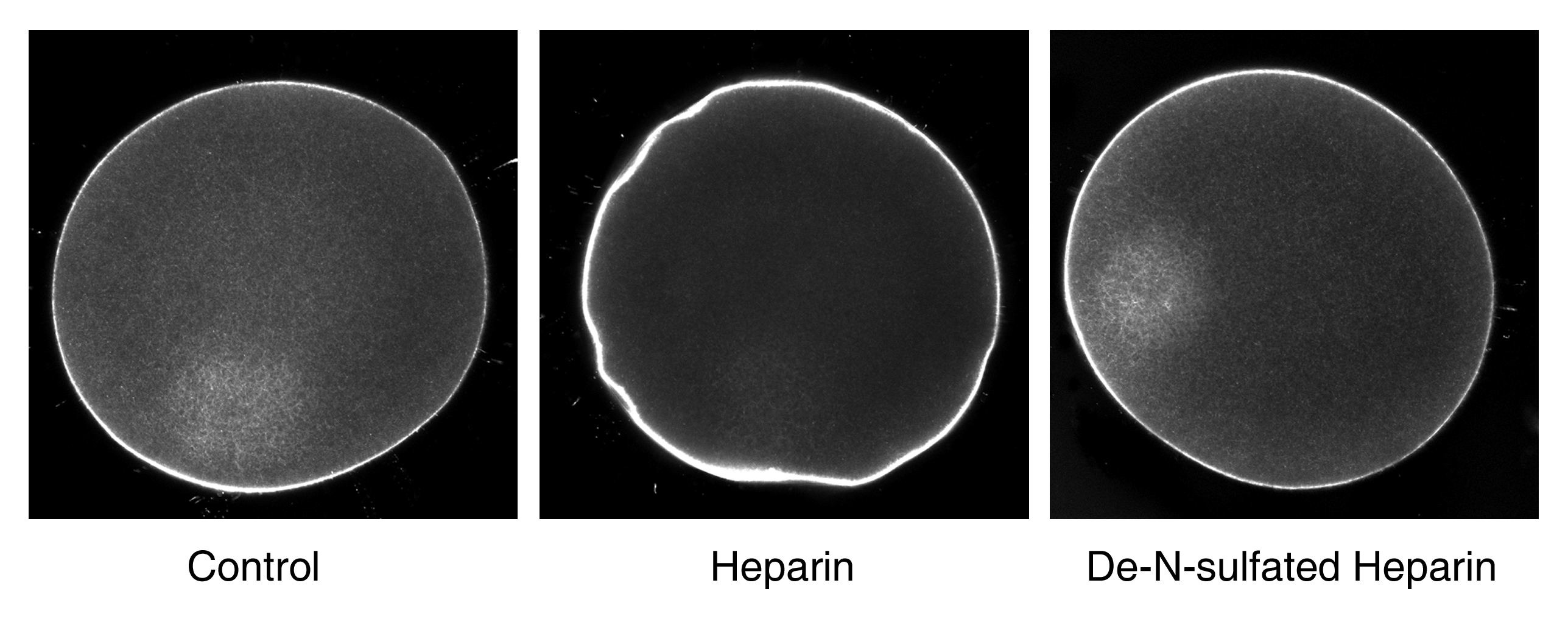

Supplement: Data S2 — Heparin hyperpolymerizes cortical actin. A. aranciacus oocytes were treated with 1-MA for 1 h to induce meiotic maturation, and then microinjected with either buffer (A), heparin (B), or De-N-sulfated heparin (C). Following cell fixation with glutaraldehyde, F-actin was stained with fluorescent phalloidin. The hyperpolymerization of cortical actin seen in heparin-treated eggs (B) was not evident in the eggs pre-injected with the structural analog of heparin (C). (0.99 MB TIF) [file pone.0003588.s002.tif]
